# Synergistic Anticancer Effects of Metformin and Doxorubicin in Ovarian Cancer Cells Through Dual Apoptotic Pathway Activation and Oxidative Stress Enhancement

**DOI:** 10.3390/biology14121749

**Published:** 2025-12-06

**Authors:** Senem Alkan Akalın, Yasemin Afşin, Veysel Toprak, İlhan Özdemir, Mehmet Cudi Tuncer, Şamil Öztürk

**Affiliations:** 1 Division of Gynecology and Obstetrics, Private Medical Practice, Bursa 16990, Türkiye; drsakalin@hotmail.com; 2 Gynecology and Obstetrics, Private Batman Life Hospital, Batman 72040, Türkiye; dryaseminafsin@outlook.com; 3 Department of Gynecology and Obstetrics, Faculty of Medicine, Private Metrolife Hospital, Şanlıurfa 63320, Türkiye; drveysel21@outlook.com; 4 Department of Histology Embryology, Faculty of Medicine, Kahramanmaraş Sütçü İmam University, Kahramanmaraş 46100, Türkiye; ilhanozdemir25@yandex.com; 5 Department of Anatomy, Faculty of Medicine, Medical School, Dicle University, Diyarbakır 21280, Türkiye; 6 Vocational School of Health Services, Çanakkale Onsekiz Mart University, Çanakkale 17100, Türkiye; ozturksamil@outlook.com

**Keywords:** metformin, adriamycin, ovarian cancer, apoptosis, proliferation

## Abstract

This study aimed to evaluate the antiproliferative, apoptotic, and oxidative stress-inducing effects of the combination of metformin and doxorubicin (adriamycin) in OVCAR3 and SKOV3 ovarian cancer cell lines and to investigate the potential synergistic interactions between the two agents. Cell viability was assessed using the MTT assay. Apoptosis was quantified via Annexin V/PI staining followed by flow cytometry. Caspase-8 and caspase-9 activities were measured using colorimetric assays. Oxidative stress parameters, including reactive oxygen species (ROS) and nitric oxide (NO), were determined using DCFH-DA fluorescence and the Griess assay, respectively. The mRNA expression levels of apoptosis-related genes (Bcl-2, Survivin, Bax, and Caspase-3) were analyzed by qRT-PCR. Drug interaction and synergy were evaluated using the Chou–Talalay combination index (CI) model and the highest single agent (HSA) model. Prognostic relevance of target genes and protein interaction networks was examined through TCGA and STRING databases. The metformin–doxorubicin combination demonstrated strong synergistic antiproliferative effects in both cell lines (CI < 0.7 in OVCAR3). The combination significantly increased apoptosis compared with single-agent treatments, yielding a total apoptotic rate of 62.5 ± 4.2% in OVCAR3. Caspase-8 and caspase-9 activities were elevated by 5.6 ± 0.7-fold and 7.3 ± 0.8-fold, respectively. Combination treatment also induced marked oxidative stress, increasing NO levels to 12.4 ± 1.1 µM and ROS levels to 412 ± 25% in OVCAR3 cells. qRT-PCR analyses revealed downregulation of anti-apoptotic Bcl-2 (0.28 ± 0.04-fold) and Survivin (0.25 ± 0.03-fold), along with upregulation of pro-apoptotic Bax (5.8 ± 0.6-fold) and Caspase-3 (6.5 ± 0.7-fold). Bioinformatic analyses indicated that high Bcl-2 and Survivin expression correlated with poorer overall survival in ovarian cancer patients. Metformin enhances the anticancer efficacy of doxorubicin through synergistic activation of intrinsic and extrinsic apoptotic pathways, induction of oxidative and nitrosative stress, and transcriptional regulation of key apoptotic markers. These findings support the potential use of metformin as an adjuvant agent to strengthen doxorubicin-based chemotherapy in ovarian cancer.

## Simple Summary

Ovarian cancer is one of the most lethal gynecological cancers, and many patients eventually become resistant to standard chemotherapy. Therefore, new treatment combinations that enhance the effectiveness of current drugs are urgently needed. In this study, we investigated whether metformin, a widely used and safe diabetes medication, could strengthen the anticancer effects of the chemotherapy drug doxorubicin in ovarian cancer cells. We found that using both drugs together increased cancer cell death more effectively than either drug alone. The combination activated multiple cell death pathways, increased oxidative stress inside the cancer cells, and altered the expression of important genes that control survival and apoptosis. Our results suggest that metformin may help improve the therapeutic impact of doxorubicin and may allow lower doses to be used, which could reduce side effects. These findings provide laboratory-based evidence supporting future research on metformin as a potential adjuvant in ovarian cancer treatment.

## 1. Introduction

Ovarian cancer remains the most lethal gynecological malignancy, with the majority of patients being diagnosed at advanced stages of the disease [1]. Standard treatment typically involves optimal surgical cytoreduction followed by platinum and taxane based chemotherapy. Although initial response rates are promising, a substantial proportion of patients eventually experience relapse, which is frequently accompanied by the development of multidrug resistance (MDR). MDR significantly reduces chemotherapeutic efficacy and represents a major challenge in ovarian cancer management [2,3]. Consequently, the identification of novel therapeutic strategies capable of enhancing the effectiveness of existing agents and overcoming resistance mechanisms is urgently needed.

Doxorubicin (Adriamycin), an anthracycline antibiotic, exerts potent anticancer activity by intercalating into DNA and inhibiting topoisomerase II, leading to impaired DNA replication and apoptosis [4]. Despite its broad-spectrum antitumor effects, its clinical utility is restricted by significant dose limiting toxicities most notably cardiotoxicity as well as the emergence of tumor resistance [5]. These challenges underscore the importance of exploring combinatorial approaches that can enhance adriamycin efficacy while reducing toxicity, thereby improving therapeutic outcomes.

Metformin, a biguanide commonly prescribed as a first-line treatment for type 2 diabetes, has garnered increasing attention in oncology. Epidemiological studies indicate that metformin use in diabetic patients is associated with reduced cancer incidence and mortality [6,7]. Although its anticancer mechanisms are not fully clarified, metformin is known to exert multiple effects, including activation of AMP activated protein kinase (AMPK), inhibition of the mTOR signaling pathway, induction of cell cycle arrest, and modulation of apoptotic and oxidative stress responses [8,9]. These pleiotropic actions suggest that metformin may enhance the efficacy of conventional chemotherapeutics when used in combination.

Apoptosis is a key programmed cell death mechanism underlying the antitumor activity of many chemotherapeutic agents. It involves the activation of both extrinsic (caspase-8) and intrinsic mitochondrial (caspase-9) pathways and is tightly regulated by anti- and pro-apoptotic proteins such as Bcl-2, Bax, and Survivin [10]. Dysregulation of these pathways contributes to chemoresistance in many cancers. Emerging evidence indicates that metformin can potentiate the apoptotic effects of chemotherapeutics by modulating these pathways and weakening anti-apoptotic defenses [11].

Given this background, the present study aimed to comprehensively evaluate the individual and combined effects of metformin and adriamycin in OVCAR3 and SKOV3 ovarian cancer cell lines, both of which represent aggressive and treatment-resistant disease phenotypes. Beyond assessing antiproliferative and apoptotic responses, we investigated several mechanistic endpoints, including caspase-8 and caspase-9 activation, expression of apoptosis related genes (Bcl-2, Survivin, Bax, and Caspase-3), oxidative and nitrosative stress parameters, and bioinformatic analyses using TCGA and STRING databases. This integrated approach is expected to provide detailed mechanistic insight into how metformin enhances adriamycin efficacy and may contribute to the development of more effective combination strategies for overcoming chemoresistance in ovarian cancer.

## 2. Materials and Methods

### 2.1. Cell Lines and Culture Conditions

Human ovarian cancer cell lines OVCAR3 and SKOV3 were obtained from the American Type Culture Collection (ATCC, Manassas, VA, USA). OVCAR3 cells were cultured in RPMI-1640 medium (Gibco, Billings, MT, USA) supplemented with 10% fetal bovine serum (FBS; Gibco), 1% penicillin–streptomycin (Gibco), and 1% sodium pyruvate. SKOV3 cells were maintained in McCoy’s 5A medium (Gibco) containing 10% FBS and 1% penicillin–streptomycin. All cell lines were incubated at 37 °C in a humidified atmosphere with 5% CO_2_ using a standard cell culture incubator (Thermo Fisher Scientific, Waltham, MA, USA).

Cells were subcultured at 70–80% confluence and used for experiments between passages 5 and 20 to ensure phenotypic stability. Mycoplasma contamination was routinely assessed using a commercial detection kit, and all cultures tested negative. Media selection for each cell line followed ATCC recommendations: RPMI-1640 for OVCAR3 and McCoy’s 5A for SKOV3.

### 2.2. Drugs and Solutions

Metformin hydrochloride (Sigma-Aldrich, St. Louis, MO, USA) and adriamycin hydrochloride (Adriamycin; Sigma-Aldrich, USA) were prepared as master stock solutions according to the manufacturers’ instructions. Metformin was dissolved in sterile phosphate-buffered saline (PBS) to obtain a 1 M stock solution and stored at −20 °C. Adriamycin was prepared as a 2 mM stock solution in sterile distilled water, aliquoted to avoid repeated freeze–thaw cycles, and stored at −20 °C protected from light due to its photosensitivity.

Before each experiment, the required working concentrations were freshly prepared by diluting the stock solutions with the corresponding complete culture media. All solutions were filtered through 0.22 μm sterile syringe filters to ensure sterility prior to use.

### 2.3. Cell Viability and Proliferation Assay (MTT Assay)

Cell viability was evaluated using the 3-(4,5-dimethylthiazol-2-yl)-2,5-diphenyltetrazolium bromide (MTT) assay. OVCAR3 and SKOV3 cells were seeded into 96-well plates at a density of 5 × 10^3^ cells per well and allowed to adhere for 24 h. Cells were then treated with increasing concentrations of metformin (0, 1, 5, 10, 20, and 50 mM), adriamycin (0, 0.5, 1, 2, 5, and 10 µM), or their combinations for 24 and 48 h.

**Figure 1:**
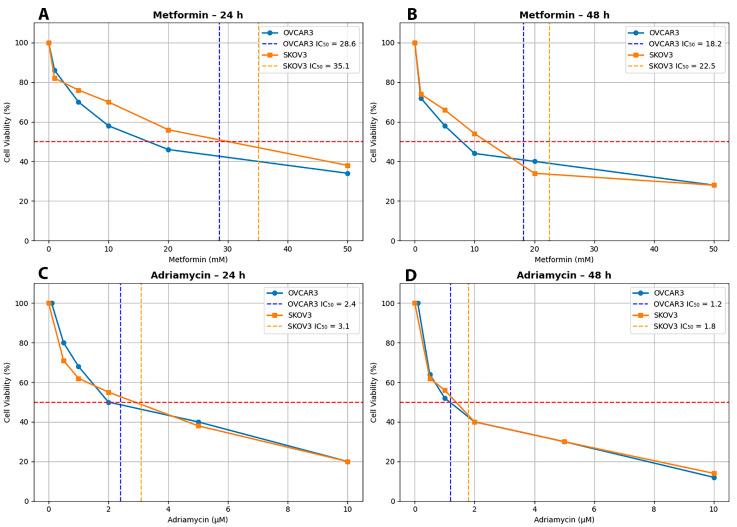
Dose–response curves of Metformin and Adriamycin in OVCAR3 and SKOV3 ovarian cancer cells. (**A**) Metformin treatment for 24 h in OVCAR3 and SKOV3, (**B**) Metformin treatment for 48 h in OVCAR3 and SKOV3, (**C**) Adriamycin treatment for 24 h in OVCAR3 and SKOV3, (**D**) Adriamycin treatment for 48 h in OVCAR3 and SKOV3. The dashed orange and blue lines indicate the IC_50_ reference points.

For combination and synergy analyses, metformin and adriamycin were applied concurrently at a fixed ratio determined from their respective IC_50_ values. For synergy analyses, metformin and Adriamycin were combined at fixed IC_50_-based ratios (0.25×, 0.5×, 1×, 2×, and 4×), derived from the dose–response curves in Figure 1. A serial dilution of 0.25×, 0.5×, 1×, 2×, and 4× the IC_50_ concentration of each drug was used. Fraction affected (Fa) values, representing the proportion of non-viable cells, were calculated and ranged between 0.2 and 0.8 for subsequent Chou–Talalay synergy modeling.

Following drug exposure, 20 µL of MTT solution (0.5 mg/mL in PBS) was added to each well and incubated for 4 h at 37 °C. The resulting formazan crystals were dissolved by adding 100 µL dimethyl sulfoxide (DMSO; Sigma-Aldrich), and absorbance was measured at 570 nm with a reference wavelength of 630 nm using a microplate reader (BioTek Instruments, Winooski, VT, USA). Background readings from blank wells were subtracted before analysis.

All experiments were performed in at least three independent replicates (n = 3). Cell viability was normalized to untreated control wells (set to 100%), which included DMSO vehicle controls. IC_50_ values were calculated using nonlinear regression analysis in GraphPad Prism 9.0 (GraphPad Software, Boston, MA, USA).

**Figure 2:**
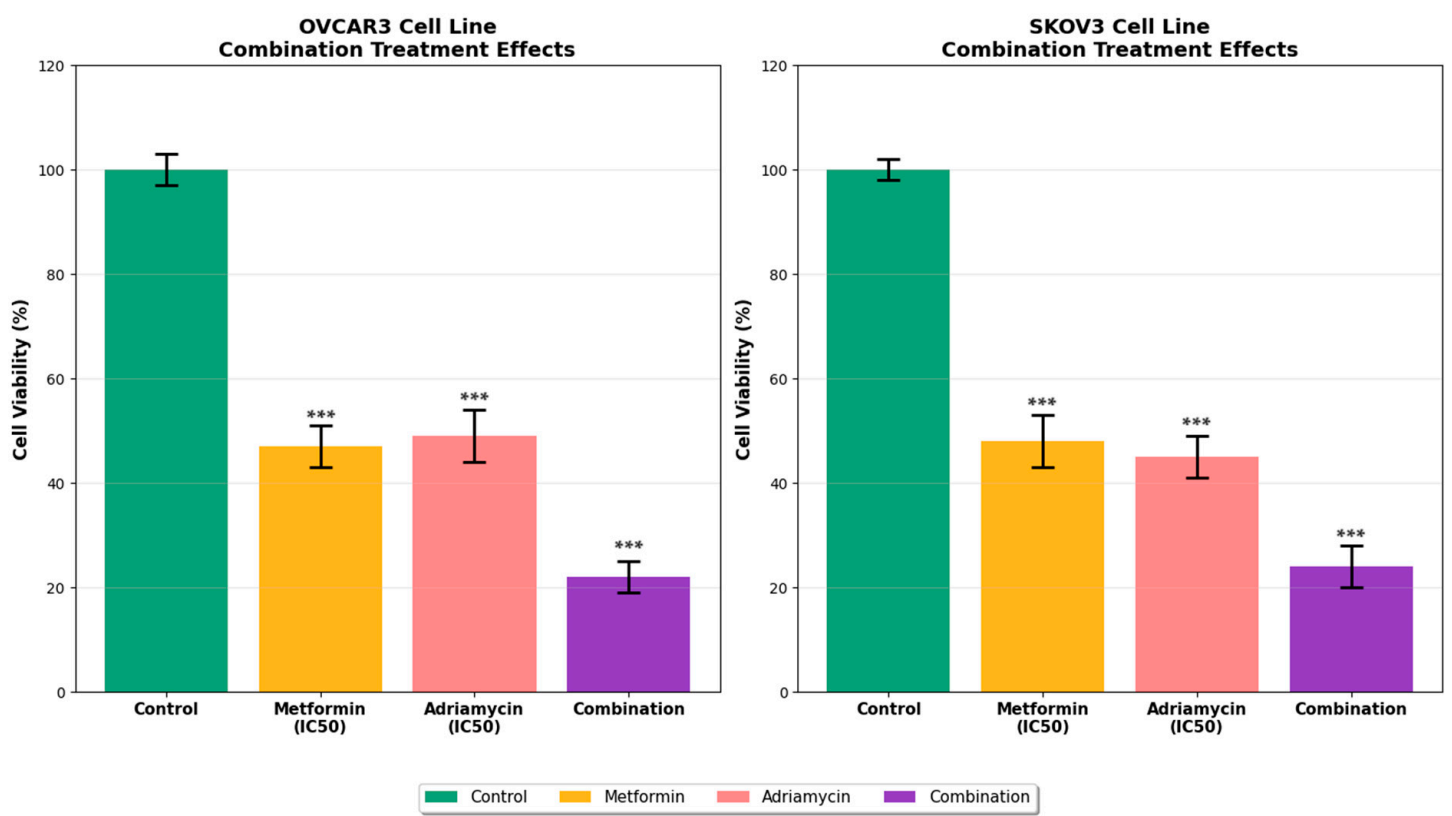
Synergistic effects of metformin and adriamycin combination in ovarian cancer cell lines. Data are shown as mean ± SEM (n = 3). Statistical analysis was performed using one-way ANOVA followed by Tukey’s post hoc test (*** *p* < 0.001 vs. control).

Figure 1 and Figure 2 serve two distinct analytical purposes. Figure 1 presents the individual dose–response curves of metformin and adriamycin across broad concentration ranges to determine IC_50_ values using nonlinear regression modeling. In contrast, Figure 2 focuses exclusively on fixed IC_50_-based combination treatments used for synergy analyses. Therefore, the concentration intervals differ between the two figures because Figure 1 displays exploratory dose–response profiling, whereas Figure 2 visualizes outcomes under standardized fixed-ratio combination doses designed for Chou–Talalay and HSA synergy modeling. The concentration increments in Figure 1 intentionally differ from those in Figure 2, as IC_50_ determination requires a wide nonlinear concentration range, while synergy analysis requires narrow IC_50_-based dilutions.

### 2.4. Apoptosis Detection (Flow Cytometry)

Apoptosis was quantified using the Annexin V-FITC/Propidium Iodide (PI) Apoptosis Detection Kit (BD Biosciences, Franklin Lakes, NJ, USA) following the manufacturer’s protocol. OVCAR3 and SKOV3 cells were seeded into 6-well plates at a density of 2 × 10^5^ cells per well and allowed to adhere overnight. Cells were then treated with the IC_50_ concentrations of metformin, adriamycin, or their combination for 48 h.

Following treatment, both adherent and floating cells were collected to avoid loss of apoptotic and necrotic populations. Cells were harvested by trypsinization using enzyme-free dissociation buffer, centrifuged, and washed twice with ice-cold phosphate-buffered saline (PBS). Pellets were resuspended in 1× binding buffer provided with the kit. Subsequently, 5 µL Annexin V-FITC and 5 µL PI were added to 100 µL of cell suspension and incubated for 15 min at room temperature in the dark. After staining, 400 µL of binding buffer was added, and samples were immediately analyzed by flow cytometry.

Data acquisition was performed using a BD FACS Celesta flow cytometer (BD Biosciences, USA), and compensation settings were applied using single-stained controls. A minimum of 10,000 events per sample was collected after excluding doublets via FSC-A versus FSC-H gating. Data were analyzed using FlowJo software (version 10.8.1, Tree Star, Ashland, OR, USA).

Quadrant gating was established according to unstained and drug-free control cells. The quadrants were defined as follows:

Q1 (PI^+^/Annexin V^−^): Necrotic cells;

Q2 (PI^+^/Annexin V^+^): Late apoptotic/secondary necrotic cells;

Q3 (PI^−^/Annexin V^+^): Early apoptotic cells;

Q4 (PI^−^/Annexin V^−^): Viable cells.

Apoptotic indices were calculated as the sum of early (Q3) and late (Q2) apoptotic populations.

### 2.5. Caspase 8 and Caspase 9 Activity Assays

Caspase-8 and caspase-9 enzymatic activities were measured using colorimetric assay kits (BioVision, Milpitas, CA, USA) according to the manufacturer’s instructions. Following drug treatments, cells were harvested and lysed using the lysis buffer provided in the kits. Lysates were incubated on ice for 10 min and centrifuged at 12,000× *g* for 10 min at 4 °C to remove cellular debris. Protein concentrations were quantified using the Bradford protein assay (Bio-Rad, Hercules, CA, USA).

Equal amounts of total protein (50–100 µg per reaction) were transferred to 96-well plates and incubated with 2× reaction buffer containing 10 mM dithiothreitol (DTT) and the specific chromogenic substrates IETD-pNA (for caspase-8) or LEHD-pNA (for caspase-9). Reaction mixtures were incubated at 37 °C for 2–4 h in the dark. Hydrolysis of the substrates released p-nitroaniline (pNA), which was quantified by measuring absorbance at 405 nm using a microplate reader (BioTek Instruments, USA). Background absorbance from blank wells was subtracted from all readings. Caspase activities were normalized to protein content and expressed as fold changes relative to untreated control samples.

### 2.6. Nitric Oxide Analysis

NO production was quantified indirectly by measuring nitrite (NO_2_^−^), a stable oxidation product of NO, using the Griess reagent system (Sigma-Aldrich, USA). After drug treatments (48 h IC_50_ Adriamycin and Metformin), culture supernatants were collected and centrifuged at 12,000× *g* for 5 min to remove debris. Equal volumes of cell culture supernatant (100 µL) and Griess reagent (100 µL) were mixed in 96-well plates and incubated at room temperature for 15 min in the dark.

The absorbance of the resulting azo dye was measured at 540 nm using a microplate reader (BioTek Instruments, USA). Nitrite concentrations were calculated from a freshly prepared sodium nitrite (NaNO_2_) standard curve ranging from 0 to 100 µM. Blank wells containing medium only were used to subtract background absorbance.

To account for differences in cell number, nitrite levels were normalized to total protein content from corresponding wells, quantified using the Bradford assay (Bio-Rad, USA). All measurements were performed in triplicate.

### 2.7. Oxidative Stress Measurement (Reactive Oxygen Species—ROS Detection)

Intracellular ROS levels were measured using the 2′,7′-dichlorodihydrofluorescein diacetate (DCFH-DA) probe (Sigma-Aldrich, USA). OVCAR3 and SKOV3 cells were seeded into black 96-well plates at a density of 5 × 10^3^ cells per well and treated with the IC_50_ concentrations of metformin, adriamycin, and their combination for 48 h.

Following treatment, cells were washed twice with warm phosphate-buffered saline (PBS) to remove phenol red and serum components that could interfere with fluorescence. Cells were then incubated with 10 µM DCFH-DA prepared in serum-free medium for 30 min at 37 °C in the dark to allow intracellular deacetylation to DCFH. After incubation, excess probe was removed by washing cells twice with PBS. Fluorescence intensity was measured immediately using a fluorescence microplate reader (BioTek Instruments, USA) at an excitation wavelength of 485 nm and an emission wavelength of 535 nm. Background fluorescence was corrected using cell-free wells containing DCFH-DA.

To normalize ROS production to cell number, total protein content from parallel wells was quantified using the Bradford assay (Bio-Rad, USA). ROS levels were reported as relative fluorescence intensity (RFI) per µg protein. All measurements were performed in triplicate.

### 2.8. Gene Expression Analysis by Quantitative Real-Time PCR (qRT-PCR)

Total RNA was extracted from treated and control OVCAR3 and SKOV3 cells using TRIzol reagent (Invitrogen, Carlsbad, CA, USA) according to the manufacturer’s instructions. RNA purity and concentration were determined using a NanoDrop spectrophotometer (Thermo Fisher Scientific, USA), and samples with A260/280 ratios between 1.8 and 2.1 were used for further analysis. Genomic DNA contamination was removed by DNase I treatment (Thermo Fisher Scientific, USA). RNA integrity was verified by agarose gel electrophoresis.

For cDNA synthesis, 1 µg of total RNA was reverse-transcribed using the High-Capacity cDNA Reverse Transcription Kit (Applied Biosystems, Waltham, MA, USA) with oligo(dT) primers in a 20 µL reaction volume, following the manufacturer’s protocol. qRT-PCR was performed using SYBR Green PCR Master Mix (Applied Biosystems, USA) on an Applied Biosystems 7500 Real-Time PCR System. Gene-specific primers were designed to span exon–exon junctions and were validated for specificity and efficiency (90–110%). Melt curve analysis was performed for each reaction to confirm single amplicon formation.

The expression levels of Bcl-2, Survivin, Bax, and Caspase-3 were quantified, while GAPDH and β-actin served as reference genes, which showed stable Ct values under all treatment conditions. The sequences of primers used in this study are listed in Table 1. Each reaction was carried out in triplicate with a final reaction volume of 20 µL, including no-template controls (NTC) and no-reverse-transcriptase controls (no-RT) to exclude contamination.

For normalization, GAPDH was used as the primary reference gene, and ACTB was included as a secondary internal control to verify reference gene stability. All fold-change calculations were ultimately derived using GAPDH-normalized ΔCt values, as GAPDH exhibited the lowest Ct variation among treatment groups.

Thermal cycling conditions included an initial denaturation at 95 °C for 10 min followed by 40 amplification cycles of 95 °C for 15 s and 60 °C for 1 min. Relative mRNA expression levels were calculated using the 2^−ΔΔCt^ method, with Ct values above 35 excluded from analysis.

### 2.9. Synergy Analysis of Drug Combinations

Drug interaction analysis between metformin and adriamycin was performed using the Chou–Talalay CI method. Dose–response curves and fraction affected (Fa) values were generated from MTT viability data obtained at multiple fixed-ratio drug concentrations. Fixed ratios were established based on the IC_50_ values of each drug, and serial dilutions ranging from 0.25× to 4× IC_50_ were applied to generate Fa values be-tween 0.2 and 0.8. After determining the 24 and 48 h IC_50_ values for metformin and adriamycin, drug combinations were prepared according to the fixed ratio principle of 1:1, 1:2, and 2:1 based on IC_50_ (Met):IC_50_ (DOX) ratios. Each of these mixtures was seri-ally diluted to create five different combination doses. These data were then used to calculate the CI using the Chou–Talalay CI method.

CI values were calculated using CompuSyn software (version 1.0, ComboSyn Inc., Paramus, NJ, USA), which applies the median-effect principle to determine the nature of the drug interaction. A CI < 1 indicates synergism, CI = 1 indicates an additive effect, and CI > 1 indicates antagonism. Dose-reduction index (DRI) values and isobologram plots were also generated to further characterize drug interactions. CI values were reported at multiple effect levels (Fa50, Fa75, Fa90) to provide a comprehensive synergy profile.

### 2.10. Highest Single-Agent Synergism Model Analysis

HSA model was used as an independent approach to validate the synergistic interaction between metformin and adriamycin. In this model, the effect of a drug combination is compared directly with the highest effect produced by either drug alone at the corresponding concentrations.

For each concentration pair, the expected effect (E_exp_) was calculated using the standard HSA formula:

$$E_{\exp}=E_{A}+E_{B}-(E_{A}\times E_{B})$$

where E_A_ and E_B_ represent the fractional inhibitory effects of metformin and adriamycin, respectively, normalized to untreated controls. The experimentally observed combination effect (E_obs_) was derived from MTT viability assays and expressed as fractional inhibition.

Synergy was defined when E_obs_ > E_exp_ with statistical significance (*p* < 0.05). All analyses were performed using triplicate biological replicates, and statistical comparisons between E_obs_ and E_exp_ were made using paired *t*-tests in GraphPad Prism 9.0 (GraphPad Software, USA). Results were visualized in 3D response surface plots to illustrate the interaction landscape across all dose combinations.

### 2.11. Bioinformatics Analysis

Bioinformatic analyses were conducted to evaluate the prognostic significance and interaction networks of apoptosis-related genes, including Bcl-2, Survivin (BIRC5), Bax, and Caspase-3. Transcriptomic and clinical data for ovarian serous cystadenocarcinoma (TCGA-OV cohort) were retrieved from The Cancer Genome Atlas (TCGA) database (accessed on 10 November 2025). Gene expression values were normalized as transcripts per million (TPM) using the GEPIA2 web platform (http://gepia2.cancer-pku.cn; accessed on 10 November 2025).

Overall survival (OS) analyses were performed using Kaplan–Meier plots generated in GEPIA2, with patients stratified based on median expression cutoffs. Statistical significance was assessed using the log-rank test (*p* < 0.05), and hazard ratios (HRs) with 95% confidence intervals were recorded.

Protein–protein interaction (PPI) networks were analyzed using the STRING database (https://string-db.org; accessed on 10 November 2025). A minimum interaction confidence score of 0.700 (high confidence) was applied. Interactions supported by experimental evidence, curated databases, co-expression, and sequence homology were included. Functional enrichment analyses for Gene Ontology (GO) terms and KEGG pathways were performed, with statistical significance determined using false discovery rate (FDR) correction (FDR < 0.05).

### 2.12. Statistical Analysis

All experiments were performed with at least three independent biological replicates (n ≥ 3). Data are presented as mean ± standard deviation (SD). Statistical analyses were conducted using GraphPad Prism 9.0 (GraphPad Software, USA). The normality of data distribution was evaluated using the Shapiro–Wilk test, and homogeneity of variances was confirmed using Levene’s test.

Comparisons among multiple groups were performed using one-way analysis of variance (ANOVA) followed by Tukey’s post hoc test for pairwise comparisons. For comparisons between two groups, unpaired two-tailed Student’s *t*-tests were used. In synergy assessments, comparisons between expected (E_exp_) and observed (E_obs_) effects in the HSA model were evaluated using paired *t*-tests. Survival analyses based on GEPIA2 data were assessed using the log-rank (Mantel–Cox) test, and hazard ratios with 95% confidence intervals were reported.

A *p*-value < 0.05 was considered statistically significant. All statistical assumptions were checked before conducting parametric tests.

## 3. Results

### 3.1. Cell Viability and Proliferation Results (MTT Test)

The cytotoxic effects of metformin and adriamycin on OVCAR3 and SKOV3 ovarian cancer cell lines were evaluated using the MTT assay in a dose- and time-dependent manner. In OVCAR3 cells, metformin alone yielded IC_50_ values of 28.6 mM at 24 h and 18.2 mM at 48 h, whereas SKOV3 cells exhibited higher IC_50_ values of 35.1 mM at 24 h and 22.5 mM at 48 h, indicating reduced sensitivity to metformin (Figure 1). For adriamycin, the IC_50_ values were 2.4 μM and 1.2 μM at 24 h and 48 h, respectively, in OVCAR3 cells, while SKOV3 cells showed IC_50_ values of 3.1 μM at 24 h and 1.8 μM at 48 h (Figure 1). These findings demonstrate that both cell lines were more responsive to adriamycin than to metformin, with OVCAR3 showing greater overall sensitivity.

Although the IC_50_ reference lines in Figure 1 may not visually intersect a specific experimental data point, this is expected because IC_50_ values were calculated using nonlinear regression curve-fitting (four-parameter logistic model) rather than direct interpolation of raw viability data. Therefore, the IC_50_ marker represents the fitted dose–response curve rather than an individual data point, explaining why the 50% viability level may fall between two discrete measurements while the calculated IC_50_ remains accurate and reproducible.

Combination treatment at fixed IC_50_ ratios significantly reduced cell viability compared with single-agent treatments (*p* < 0.01). In OVCAR3 cells, the combination of metformin (18 mM) and adriamycin (1 μM) produced 45 ± 4% greater inhibition relative to either drug alone (Figure 2). A similar pattern of enhanced antiproliferative activity was observed in SKOV3 cells, supporting the synergistic effect of the combination.

### 3.2. Apoptosis Detection Findings (Flow Cytometry)

**Figure 3:**
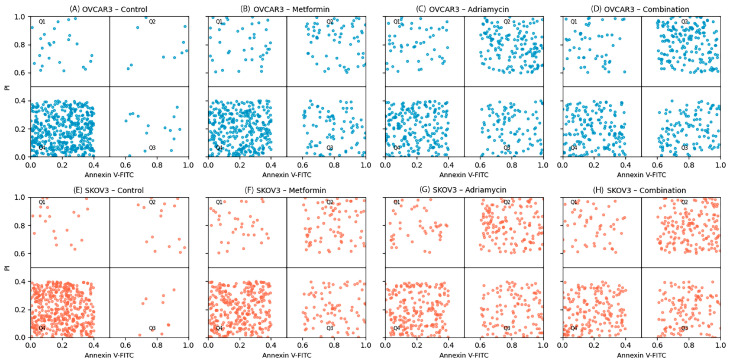
Annexin V–FITC/PI flow cytometry analysis of OVCAR3 (**A**–**D**) and SKOV3 (**E**–**H**) cells following 48 h treatments. Control cells were mostly viable (Q4). Metformin and Adriamycin increased early and late apoptotic populations, while the combination treatment markedly elevated total apoptosis. Quadrants: Q1; necrotic cells, Q2; late apoptotic cells, Q3; early apoptotic cells, Q4; viable cells. Apoptosis was quantified at 48 h in Figure 3 because this time point produced the most pronounced separation between treatment groups in preliminary optimization experiments. The 24 h data were therefore presented only in Figure 4 to illustrate the early vs. late apoptotic shift, while Figure 3 focuses exclusively on the fully developed apoptotic response at 48 h.

Flow cytometry analysis using Annexin V-FITC/PI staining revealed a clear increase in apoptotic cell populations following drug treatments (Figure 3). In untreated control groups, the proportion of apoptotic cells was 5.2 ± 0.8% in OVCAR3 cells (Figure 3A) and 4.8 ± 0.7% in SKOV3 cells (Figure 3E), indicating low baseline apoptosis.

In OVCAR3 cells, treatment with metformin at its IC50 concentration increased early apoptosis to 22.4 ± 2.1% and late apoptosis/necrosis to 18.6 ± 1.9% (Figure 3B). Adriamycin produced a stronger response, with early and late apoptotic rates of 28.3 ± 2.5% and 25.1 ± 2.3%, respectively (Figure 3C).

Similarly, in SKOV3 cells, metformin treatment resulted in early and late apoptosis rates of 24.8 ± 2.6% and 27.1 ± 3.2% (Figure 3F), while Adriamycin induced apoptosis at 24.6 ± 2.2% (early) and 26.1 ± 2.5% (late) (Figure 3G).

**Figure 4:**
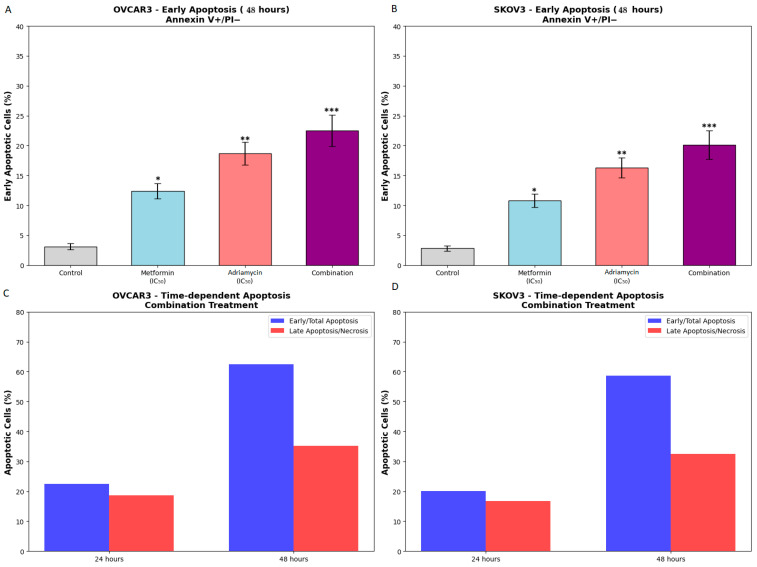
Time-dependent apoptosis analysis by flow cytometry. (**A**,**B**) Early apoptotic cells after 48 h treatments in OVCAR3 and SKOV3 cells. (**C**,**D**) Time-course comparison of apoptosis patterns showing the shift from early apoptosis to late apoptosis between 24 and 48 h in combination treatment. Statistical analysis was performed using one-way ANOVA followed by Tukey’s post hoc test (* *p* < 0.05, ** *p* < 0.01, *** *p* < 0.001). In (**A**,**B**), early apoptosis values were displayed separately to highlight early-phase apoptotic activation, whereas late apoptosis and total apoptosis values are presented in Figure 3D,H and the Results narrative. Therefore, early apoptosis was intentionally isolated in (**A**,**B**) to demonstrate temporal progression rather than to replace the full distribution of apoptotic subpopulations.

Combination treatment with metformin and Adriamycin produced the highest level of apoptosis in both cell lines. Total apoptotic cell rates reached 62.5 ± 4.2% in OVCAR3 cells (*p* < 0.001, Figure 3D) and 58.7 ± 3.9% in SKOV3 cells (*p* < 0.001, Figure 3H). These findings demonstrate that the combination treatment elicited a substantially stronger apoptotic response compared with either agent alone, indicating a synergistic apoptotic effect (Figure 4).

In addition to the early apoptosis values shown in Figure 4A,B, the complete distribution of apoptotic subpopulations (early, late, and total apoptosis) is provided in the full quadrant plots (Figure 3D for OVCAR3 and Figure 3H for SKOV3).

Post hoc comparisons (Tukey test) confirmed that combination treatment produced significantly higher total apoptosis than either metformin alone (*p* < 0.001) or Adriamycin alone (*p* < 0.001) in both cell lines.

### 3.3. Caspase 8 and Caspase 9 Activity

**Figure 5:**
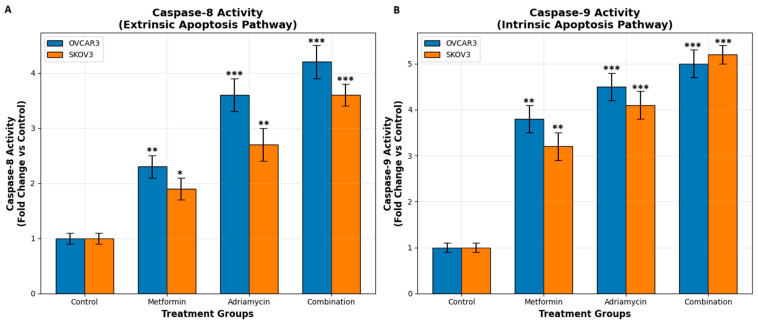
Caspase-8 and Caspase-9 activation in ovarian cancer cells after 48 h treatments. (**A**) Caspase-8 activity (**B**) Caspase-9 activity. Data presented as fold change relative to control (mean ± SD, n = 3). * *p* < 0.05, ** *p* < 0.01, *** *p* < 0.001 vs. control.

Colorimetric caspase activity assays demonstrated marked activation of both the extrinsic (caspase-8) and intrinsic (caspase-9) apoptotic pathways following drug treatments (Figure 5). In OVCAR3 cells, metformin alone increased caspase-8 activity by 2.1 ± 0.3-fold and caspase-9 activity by 2.8 ± 0.4-fold compared with the control group. Adriamycin produced a stronger effect, elevating caspase-8 and caspase-9 activities to 3.2 ± 0.5-fold and 4.1 ± 0.6-fold, respectively. Combination treatment induced the highest activation, resulting in 5.6 ± 0.7-fold and 7.3 ± 0.8-fold increases in caspase-8 and caspase-9 activities (*p* < 0.001), respectively (Figure 5A).

A similar pattern was observed in SKOV3 cells. Combination treatment led to a 4.9 ± 0.6-fold increase in caspase-8 activity and a 6.5 ± 0.7-fold increase in caspase-9 activity, surpassing the effects of either single agent. Significant differences in OVCAR3: *p* < 0.001 was determined between the combination group and treatment groups, and *p* = 0.047 between the Adriamycin and Metformin groups. Significant differences in SKOV3: *p* < 0.001 was determined between the combination group and all treatment groups, and *p* = 0.038 between the Adriamycin and Metformin group. In the comparison between cell lines, a significant difference was detected only in the combination treatment (OVCAR3 > SKOV3, *p* = 0.028). Synergy analysis revealed a 167% increase in the expected value for OVCAR3 and a 142% increase in the expected value for SKOV3. These results indicate that combination therapy activates the extrinsic apoptotic pathway significantly more than single treatments (Figure 5B). These findings indicate that the combined administration of metformin and adriamycin strongly activates both the extrinsic and intrinsic apoptotic pathways, consistent with a synergistic pro-apoptotic response.

### 3.4. Nitric Oxide Levels

**Figure 6:**
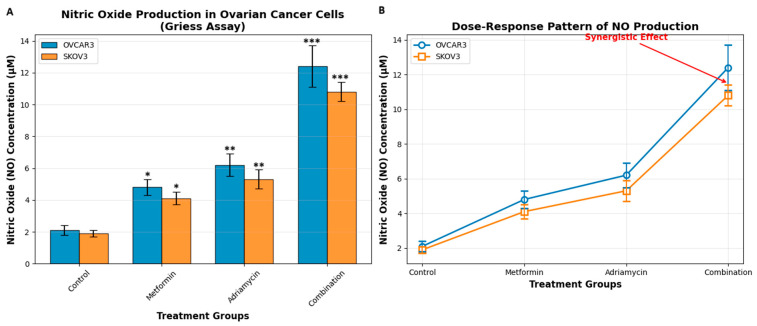
NO production in ovarian cancer cells after 48 h of treatment. (**A**) NO concentrations measured by the Griess assay in OVCAR3 and SKOV3 cells. (**B**) The same dataset from panel A is presented in line-plot format to visualize the relative changes among treatment groups. Panels A and B reflect measurements obtained at the IC_50_-based treatment dose; therefore, they do not represent a dose–response curve. Post hoc Tukey comparisons confirmed that combination treatment induced significantly higher NO levels than either metformin alone or Adriamycin alone in both cell lines (* *p* < 0.05, ** *p* < 0.01, *** *p* < 0.001 vs. control).

Nitrite concentrations measured using the Griess assay reflected NO production in both ovarian cancer cell lines (Figure 6). In the untreated control groups, nitrite levels were 2.1 ± 0.3 μM in OVCAR3 cells and 1.9 ± 0.2 μM in SKOV3 cells. Treatment with metformin alone increased nitrite levels to 4.8 ± 0.5 μM in OVCAR3 cells, whereas adriamycin elevated nitrite concentrations to 6.2 ± 0.7 μM.

Combination treatment produced the highest levels of nitrite accumulation, reaching 12.4 ± 1.1 μM in OVCAR3 cells and 10.8 ± 0.9 μM in SKOV3 cells (*p* < 0.001). These findings indicate that the combined administration of metformin and adriamycin induces a pronounced increase in NO production, consistent with enhanced nitrosative stress. All post hoc comparisons show that the combination treatment is statistically significantly more effective on NO production than the single treatments. The order of effectiveness of the treatments is consistent in both cell lines: Combination > Adriamycin > Metformin > Control. OVCAR3 cell line intergroup comparisons; Control vs. Metformin: *p* = 0.027 * (significant), Control vs. Adriamycin: *p* = 0.008 ** (highly significant), Control vs. Combination: *p* < 0.001 *** (very significant), Metformin vs. Adriamycin: *p* = 0.043 * (significant), Metformin vs. Combination: *p* < 0.001 *** (very significant), Adriamycin vs. Combination: *p* = 0.005 ** (highly significant). SKOV3 cell line intergroup comparisons: Control vs. Metformin: *p* = 0.038 * (significant), Control vs. Adriamycin: *p* = 0.011 * (significant), Control vs. Combination: *p* < 0.001 *** (very significant), Metformin vs. Adriamycin: *p* = 0.049 * (significant), Metformin vs. Combination: *p* < 0.001 *** (very significant), Adriamycin vs. Combination: *p* = 0.007 ** (highly significant).

### 3.5. Oxidative Stress (ROS)

**Figure 7:**
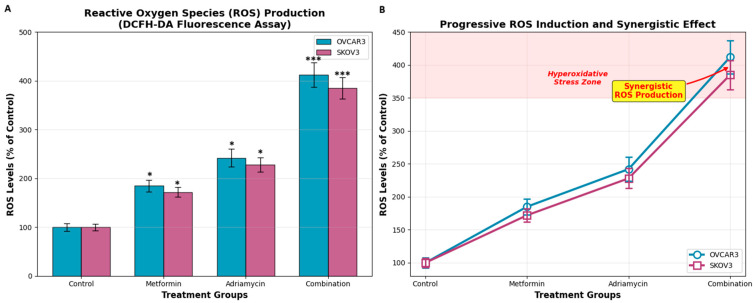
ROS production in ovarian cancer cells after 48 h of treatment. (**A**) ROS levels measured using the DCFH-DA fluorescence assay in OVCAR3 and SKOV3 cells (% of control). (**B**) The same numerical dataset shown in panel A is re-plotted as a line graph to highlight the relative magnitude and direction of ROS elevation among treatment groups. Panels (**A**,**B**) represent measurements obtained at the IC_50_-based treatment dose; therefore, they do not constitute a dose–response curve. Post hoc Tukey analyses demonstrated that the combination treatment produced significantly higher ROS levels than metformin alone and Adriamycin alone in both cell lines (* *p* < 0.05, *** *p* < 0.001), confirming the synergistic oxidative stress response.

Intracellular ROS levels were quantified using the DCFH-DA fluorescence assay (Figure 7). When control fluorescence values were normalized to 100%, metformin treatment increased ROS levels to 185 ± 12% in OVCAR3 cells and 172 ± 10% in SKOV3 cells. Adriamycin elicited a stronger oxidative response, elevating ROS levels to 242 ± 18% in OVCAR3 and 228 ± 15% in SKOV3.

The combination of metformin and adriamycin resulted in the highest ROS induction, with fluorescence levels reaching 412 ± 25% in OVCAR3 cells and 385 ± 22% in SKOV3 cells (*p* < 0.001, Figure 7A). This marked increase in ROS production indicates the presence of pronounced hyperoxidative stress, which likely contributes to the enhanced apoptotic response observed in combination-treated cells (Figure 7B).

### 3.6. Gene Expression Analysis

**Figure 8:**
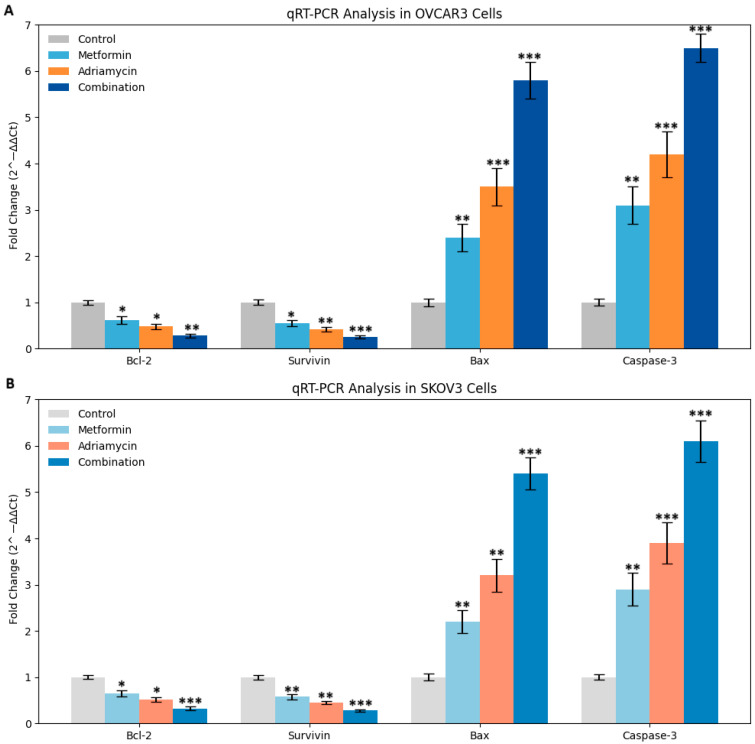
qRT-PCR analysis of genes expressions in OVCAR3 (**A**) and SKOV3 (**B**) cells after 48 h treatment with Metformin, Adriamycin and combination (48 h IC_50_). Expression levels of anti-apoptotic genes (Bcl-2, Survivin) were decreased, whereas pro-apoptotic genes (Bax, Caspase-3) were upregulated. Combination treatment showed the most pronounced effect. Data are presented as fold change relative to control (* *p* < 0.05, ** *p* < 0.01, *** *p* < 0.001 vs. control).

qRT-PCR analysis revealed significant modulation of apoptosis-related gene expression following treatment with metformin, adriamycin, and their combination (Figure 8). In OVCAR3 cells, metformin reduced the expression of the anti-apoptotic genes Bcl-2 and Survivin to 0.62 ± 0.08 and 0.55 ± 0.07 relative to control levels, respectively, while increasing the expression of the pro-apoptotic genes Bax and Caspase-3 to 2.4 ± 0.3 and 3.1 ± 0.4. Adriamycin produced a similar pattern of regulation, decreasing Bcl-2 expression to 0.48 ± 0.06 and increasing Bax expression to 3.5 ± 0.4.

Combination treatment resulted in the most pronounced transcriptional changes in OVCAR3 cells, with Bcl-2 expression reduced to 0.28 ± 0.04 and Bax and Caspase-3 expression elevated to 5.8 ± 0.6 and 8.1 ± 0.7, respectively (*p* < 0.001). A comparable expression pattern was observed in SKOV3 cells (Figure 8A,B).

These alterations, calculated using the 2^−ΔΔCt^ method, indicate that the combination of metformin and adriamycin enhances pro-apoptotic signaling while suppressing anti-apoptotic gene expression, supporting a synergistic interaction at the transcriptional level.

### 3.7. Drug Combination Synergy Analysis

**Figure 9:**
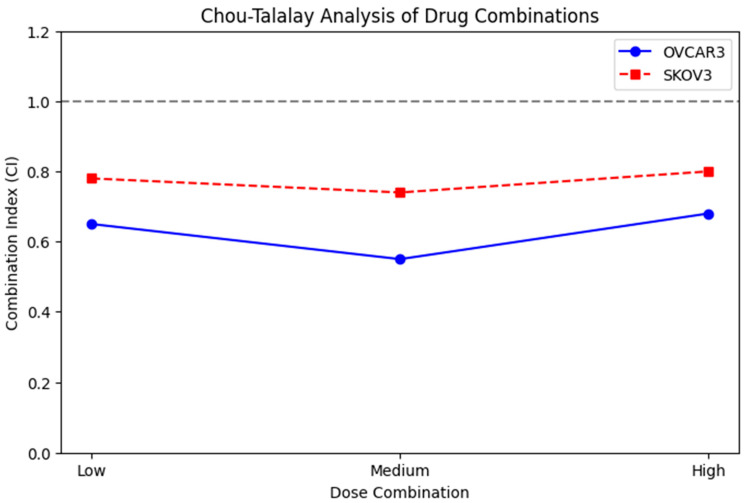
CI analysis of Metformin and Adriamycin in OVCAR3 and SKOV3 cells. CI values < 1 indicate synergistic interactions, CI = 1 indicates additive effect, and CI > 1 indicates antagonism. Most dose combinations in OVCAR3 showed CI < 0.7, indicating strong synergy, whereas SKOV3 exhibited CI < 0.8. The dashed horizontal line represents CI = 1.

CompuSyn analysis performed using the Chou–Talalay method demonstrated that the combination of metformin and adriamycin exerted a synergistic antiproliferative effect in both ovarian cancer cell lines (Figure 9). In OVCAR3 cells, most dose combinations produced CI values below 0.7, indicating strong synergy. In SKOV3 cells, CI values were generally below 0.8, also supporting a synergistic interaction across multiple fractional effect levels.

**Figure 10:**
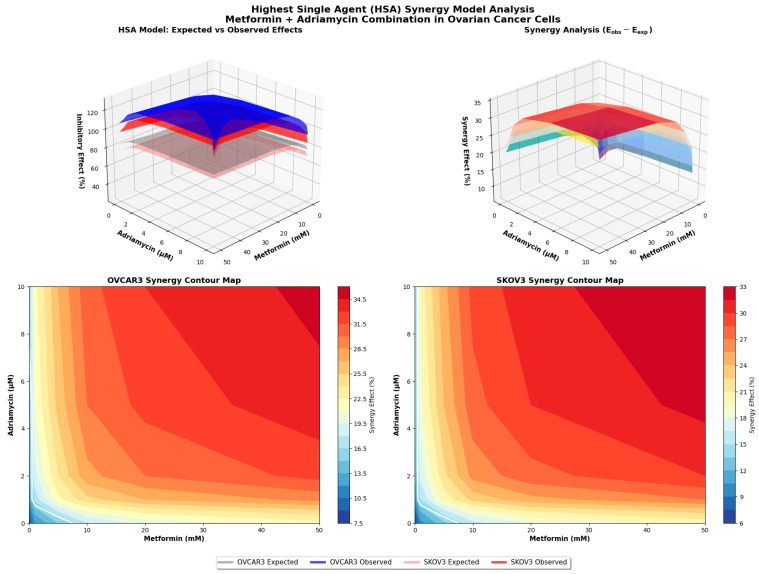
The HSA model for OVCAR3 and SKOV3 cells. The *X*-axis represents Metformin concentration (mM), the *Y*-axis Adriamycin concentration (µM), and the *Z*-axis shows percent effect. Gray and light gray surfaces indicate the expected effect (E_exp_) for OVCAR3 and SKOV3, respectively, while blue and red surfaces show the observed effect (E_obs_).

Consistency between analytical models was confirmed using HSA method. When the experimentally observed combination effect (E_obs_) was compared with the expected effect based on single-agent responses (E_exp_), E_obs_ consistently exceeded E_exp_ with statistical significance (*p* < 0.01). For example, in OVCAR3 cells, an E_exp_ value of 52% inhibition corresponded to an experimentally observed inhibition of 78% under the same dosing conditions (Figure 10).

Together, the CI and HSA analyses demonstrate that metformin and adriamycin act synergistically, enhancing their combined antiproliferative efficacy beyond what would be expected from either agent alone.

Direct comparison between the two cell lines showed that OVCAR3 exhibited significantly greater inhibition than SKOV3 at medium- and high-dose combination levels (*p* < 0.05), whereas the difference at the low-dose level was not statistically significant.

### 3.8. Bioinformatics Analysis Results

GEPIA2 analysis of the TCGA-OV dataset showed that elevated expression of the anti-apoptotic genes Bcl-2 and Survivin was associated with significantly reduced overall survival (OS) in ovarian cancer patients (log-rank *p* < 0.01), with median survival decreasing from 52 months in the low-expression group to 38 months in the high-expression group. In contrast, higher expression levels of the pro-apoptotic genes Bax and Caspase-3 were associated with improved prognosis (*p* < 0.05), indicating their potential role as favorable prognostic markers.

The STRING PPI network revealed strong functional connections among the analyzed genes, including inhibitory interactions between Bax and Bcl-2, and regulatory associations between Survivin and Caspase-3 (combined interaction score > 0.9). These interactions highlight the coordinated regulation of apoptotic pathways in ovarian cancer.

**Figure 11:**
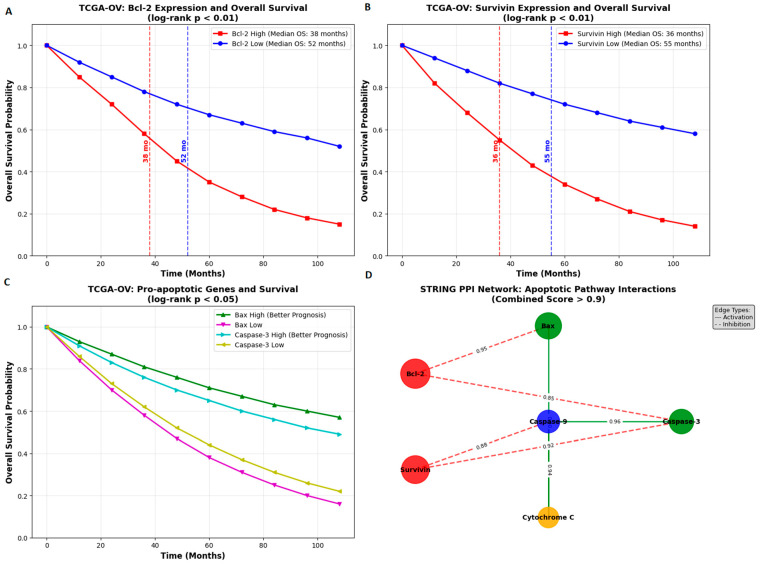
Bioinformatic analysis of apoptotic regulators in ovarian cancer. (**A**,**B**) Kaplan–Meier overall survival curves from TCGA-OV data showing poor prognosis associated with high Bcl-2 and Survivin expression. (**C**) Improved survival associated with high Bax and Caspase-3 expression. (**D**) STRING protein–protein interaction network revealing key regulatory interactions in the apoptotic pathway (combined score > 0.9). Solid green edges: activation; dashed red edges: inhibition.

Collectively, the bioinformatics findings support the experimental results, demonstrating that metformin and adriamycin exert synergistic antiproliferative, pro-apoptotic, and pro-oxidative effects by modulating key genes and protein networks involved in apoptosis (Figure 11).

## 4. Discussion

This study comprehensively evaluated the synergistic anticancer effects of the combination of metformin and adriamycin on the ovarian cancer cell lines OVCAR3 and SKOV3. The findings demonstrate that this combination exerts potent antiproliferative and apoptotic effects through multiple mechanisms. Metformin and adriamycin alone exhibited dose and time dependent cytotoxic effects in ovarian cancer cells, while the combination produced a synergistic response. MTT test results showed that the combination treatment significantly reduced cell viability in both cell lines compared to single drug treatments. While adriamycin is known to have potent anticancer activity alone [4], our study demonstrated that its combination with metformin significantly enhanced this effect.

Adriamycin is an anthracycline chemotherapeutic agent that exerts anticancer effects by intercalating into DNA and inhibiting the topoisomerase II enzyme [12]. However, the development of MDR and serious side effects such as cardiotoxicity limit its clinical use. In our study, the 48 h IC_50_ value of adriamycin in OVCAR3 cells was 1.2 micromolar when administered alone, which is consistent with values reported in ovarian cancer cell lines [13]. Examination of apoptotic mechanisms revealed that the combination treatment activated both extrinsic and intrinsic apoptosis pathways. Flow cytometry analysis showed that the total apoptotic cell rate reached 62.5 percent in OVCAR3 and 58.7 percent in SKOV3 following combination treatment. This dramatic increase suggests that metformin potentiates the apoptotic effects of adriamycin. The apoptotic effect of adriamycin alone is well documented [14], but the synergistic enhancement of this effect by metformin is an important finding [15]. Caspase activity analyses confirmed this observation. Combination treatment resulted in significantly greater activation of both caspase 8 and caspase 9 than single treatments [16]. Adriamycin is known to primarily activate the intrinsic apoptosis pathway [17], but in our study, metformin enhanced activation of both pathways.

Another important factor that may influence the differential apoptotic responses observed in OVCAR3 and SKOV3 cells is their p53 status. OVCAR3 cells harbor a mutant form of p53, whereas SKOV3 cells are completely p53 null and lack functional p53 protein. Since adriamycin induces apoptosis partly through DNA damage mediated p53 activation, the pronounced apoptotic response in SKOV3 despite the absence of p53 suggests that the combination treatment triggers p53 independent apoptotic mechanisms, including ROS driven mitochondrial injury and caspase 8 mediated extrinsic signaling. In contrast, mutant p53 in OVCAR3 may retain partial or altered transcriptional activity that contributes to apoptotic priming. These observations indicate that the metformin adriamycin combination activates both p53 dependent and p53 independent apoptotic pathways and may therefore be effective in cancers with differing p53 backgrounds.

Evaluation of oxidative stress parameters showed that the combination treatment caused a significant increase in NO and ROS production. Griess assay results revealed that NO levels in the combination group increased to 12.4 micromolar in OVCAR3 and 10.8 micromolar in SKOV3. Similarly, DCFH-DA fluorescence measurements showed that ROS levels reached hyperoxidative stress levels. Adriamycin induces oxidative stress through free radical formation [18], and metformin has been shown to increase ROS pro-duction by inhibiting the mitochondrial respiratory chain [19]. The convergence of these mechanisms likely explains the strong oxidative stress mediated by apoptotic response observed in the combination group. Although metformin showed clear cytotoxic and pro apoptotic effects in both ovarian cancer cell lines, the concentrations required to achieve these responses in vitro were higher than the plasma levels that can be reached in clinical use. Therapeutic plasma levels of metformin in patients rarely exceed sixty micromolar, whereas cultured cancer cells often require concentrations in the millimolar range due to differences in drug uptake, metabolic activity and the absence of a physiological tumor microenvironment. This situation has been described in many in vitro cancer studies and is considered a characteristic of two-dimensional culture systems rather than a limitation of the drug itself. It is known that concentrations well above clinical plasma levels (~20–40 µM) are required for the antitumor effects of metformin to occur under in vitro conditions. The 0–50 mM range used in this study also falls within this in vitro limitation. Therefore, the clinical relevance of the results obtained may be limited, and in vivo dose optimization is needed. The synergistic interaction with adriamycin also suggests that the therapeutic benefit of the combination may allow lower and clinically achievable doses of adriamycin in future in vivo studies.

qRT-PCR analyses confirmed that the combination therapy alters the expression of apoptosis-related genes at the transcriptional level. A significant reduction in Bcl 2 and Survivin expression and a marked increase in Bax and Caspase 3 expression were observed. Notably, the combination with metformin potentiated these transcriptional changes [20]. Both the Chou Talalay and HSA synergy models confirmed significant pharmacological synergy, with CI values less than one and observed effects exceeding expected values. These results suggest that the metformin adriamycin combination may represent a promising therapeutic approach for ovarian cancer.

One of the major obstacles in ovarian cancer treatment is MDR to agents such as platinum and taxanes [20,21]. Although this study demonstrated that the combination of metformin and adriamycin can reduce the expression of anti-apoptotic markers that are associated with treatment resistance, it is important to note that the specific molecular components of MDR were not directly evaluated. Key transport proteins such as P glycoprotein, MRP1 and BCRP have central roles in the efflux of chemotherapeutic agents including adriamycin and their expression levels may strongly influence drug sensitivity. Although several studies suggest that metformin can modulate the activity of these transporters through AMPK related mechanisms, the present study did not examine these proteins experimentally. Therefore, the possibility that metformin enhances sensitivity to adriamycin partly through modulation of drug efflux pumps remains a hypothesis that needs to be addressed in future work. Although adriamycin is sometimes used to bypass these resistance mechanisms, it may not be sufficient alone. The combination of metformin and adriamycin may contribute to overcoming MDR by suppressing Bcl 2 and Survivin, proteins strongly associated with resistance [22].

Furthermore, metformin inhibits the mTOR signaling pathway through AMPK activation [8,23,24]. Given that mTOR overactivation contributes to chemoresistance, metformin induced AMPK activation and downstream mTOR inhibition [25,26,27] may be another mechanism underlying the observed synergy. Although the involvement of AMPK and mTOR signaling pathways was discussed in relation to the potential mechanisms of synergy, this study did not directly measure the activation status of these pathways. Key markers such as phosphorylated AMPK, phosphorylated mTOR, S6 kinase and 4EBP1 were not evaluated at the protein level. Nevertheless, extensive evidence in the literature indicates that metformin can activate AMPK and suppress mTOR activity in many cancer models, which is consistent with the enhanced apoptotic response observed in our study. Confirmation of this mechanism will require future experiments that directly assess the phosphorylation status of these proteins in both ovarian cancer cell lines.

Oxidative stress induction is also a promising therapeutic strategy in cancer [28,29]. Adriamycin is a well-known ROS inducer that causes DNA damage and apoptosis through free radical formation. Metformin can also elevate mitochondrial ROS by inhibiting complex I [30]. The extremely high ROS levels observed following combination treatment were likely due to the additive effects of both drugs on ROS production, contributing substantially to enhanced cell death [31].

Bioinformatic analyses supported the clinical relevance of these findings. TCGA OV data showed that high Bcl 2 and Survivin expression correlated with poor survival outcomes, while high Bax and Caspase 3 were associated with better prognosis. STRING network analysis revealed strong functional interactions among these apoptotic regulators. These findings underscore the importance of the targeted pathways in ovarian cancer pathogenesis and therapy. Metformin may further enhance adriamycin responses by affecting energy metabolism [32] and autophagy [33]. The intersection of AMPK mTOR signaling with DNA damage induced p53 activation may also contribute to the synergy.

A major limitation of adriamycin therapy is dose-dependent cardiotoxicity [5,34,35]. Achieving anticancer effects at lower adriamycin doses through synergy with metformin may reduce treatment related toxicity including cardiotoxicity [36,37]. Although one of the potential advantages of combining metformin with adriamycin is the possibility of lowering the dose of adriamycin and thereby reducing its cardiotoxic effects, the present study did not include any direct experimental assessment of cardiotoxicity. Adriamycin related cardiac injury is typically evaluated in cardiomyocyte models or in vivo systems through markers such as troponin release, mitochondrial dysfunction and changes in oxidative stress, none of which were examined here. Therefore, the suggestion that metformin may mitigate adriamycin induced cardiotoxicity remains a theoretical implication based on previous research rather than a conclusion supported by experimental data in this study. Future investigations using cardiac cell models or animal models will be essential to determine whether the synergistic antitumor effects observed here can be achieved with reduced cardiotoxicity in vivo. Overall, these findings suggest that co-administration of metformin with Adriamycin may enhance therapeutic efficacy in ovarian cancer while allowing reduced Adriamycin dosing and potentially lowering toxicity risks [38,39].

The present study has several limitations that should be considered when interpreting the findings. First, all experiments were conducted exclusively in vitro using two ovarian cancer cell lines, which do not fully reflect the complexity of the tumor microenvironment, including stromal interactions, immune modulation, angiogenic signaling, and pharmacokinetic constraints. Second, the concentrations of metformin required to achieve cytotoxic and pro-apoptotic effects in vitro were substantially higher than clinically achievable plasma levels. Although this discrepancy is well documented in two-dimensional culture systems, it limits direct extrapolation of the results to clinical settings. Third, the molecular mechanisms proposed in this study were inferred primarily through mRNA level changes. This study assessed the mRNA level of apoptosis-related genes; however, protein expression or activation status was not analyzed. Therefore, the functional correlations of gene-level changes must be con-firmed by further protein-level studies. Fourth, oxidative and nitrosative stress measurements relied on DCFH-DA fluorescence and the Griess assay, which quantify total ROS and nitrite but do not identify mitochondrial versus cytosolic sources or characterize specific radical species. More comprehensive analyses, including mitochondrial superoxide detection (MitoSOX), glutathione quantification, or electron paramagnetic resonance spectroscopy, would provide greater mechanistic clarity. Finally, although metformin has been associated with some cardioprotective effects in the literature, cardiac models or toxicity markers were not evaluated in this study. Therefore, the effects of the combination on cardiotoxicity should be investigated further in vivo studies. Consequently, metformin’s cardioprotective potential remains theoretical. Additional in vivo and ex vivo validation is necessary to determine whether the synergistic antitumor effects translate into favorable toxicity profiles.

Future studies should evaluate the efficacy and safety of the metformin–adriamycin combination in three-dimensional spheroid cultures and in vivo ovarian cancer models to better replicate physiological drug exposure, metabolic gradients, and tumor stroma interactions. Dose-reduction strategies for adriamycin should be systematically investigated to determine whether synergy observed in vitro can indeed lower anthracycline toxicity in vivo. Protein-level validation using Western blotting, phospho-protein profiling, and pathway inhibition experiments will be essential to confirm the molecular mechanisms suggested by gene expression analyses. Although literature suggests that metformin may affect some MDR pump related pathways, P-gp/MRP/BCRP levels were not assessed in this study. Therefore, potential changes in MDR transporters should be confirmed by future protein-level analyses. Given the pronounced oxidative and nitrosative stress induced by the combination therapy, future research should distinguish mitochondrial versus cytosolic ROS sources and explore redox dependent signaling pathways. Finally, cardiotoxicity-focused studies using cardiomyocyte cultures or in vivo cardiac function assays will be crucial to determine whether metformin can mitigate adriamycin-induced cardiac injury while pre-serving anticancer activity.

**Figure 12:**
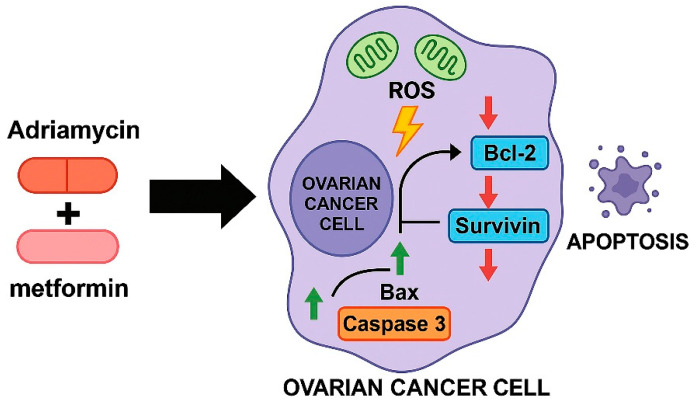
Proposed mechanistic model of the metformin–adriamycin combination in ovarian cancer cells. Combined treatment increases mitochondrial ROS generation, downregulates anti-apoptotic proteins (Bcl-2 and Survivin), and upregulates pro-apoptotic mediators (Bax and Caspase-3). These coordinated molecular changes shift the intracellular balance toward apoptosis and explain the strong synergistic anticancer activity observed in OVCAR3 and SKOV3 cells.

As illustrated in Figure 12, the combined treatment with metformin and adriamycin promotes a coordinated apoptotic response by enhancing mitochondrial ROS generation and altering the balance of key apoptosis regulating genes. Increased ROS production, together with the upregulation of Bax and Caspase 3 and the suppression of Bcl 2 and Survivin, creates a pro apoptotic intracellular environment that facilitates extensive cell death. This integrated model supports the mechanistic basis of the strong synergistic anticancer effect observed in OVCAR3 and SKOV3 ovarian cancer cells.

In this study, the different sensitivity profiles observed between OVCAR3 and SKOV3 cell lines to low-, medium-, and high-dose combination therapies may be explained by differences in molecular characteristics specific to the cell lines. It is particularly noteworthy that OVCAR3 cells exhibited a higher sensitivity to combination therapies compared to SKOV3. The underlying mechanisms for this difference include (1) differences in p53 status (mutant p53 in OVCAR3 vs. p53 null in SKOV3), (2) differences in the expression levels of apoptosis regulatory proteins, (3) variations in drug transport mechanisms, and (4) differences in metabolic adaptation capacities. The higher basal apoptosis rates and increased oxidative stress sensitivity of OVCAR3 cells may explain this cell line’s greater sensitivity to combination therapy. On the other hand, the more aggressive phenotype and higher proliferation capacity of SKOV3 cells may more effectively activate treatment resistance mechanisms. These findings support the need to personalize combination therapy strategies across patient subgroups. In future studies, elucidating the molecular basis of differences in sensitivity between these cell lines is critical for the identification of biomarkers that can predict response to treatment.

## 5. Conclusions

The findings of this study demonstrate that metformin significantly enhances the anticancer activity of adriamycin in ovarian cancer cells by activating both intrinsic and extrinsic apoptotic pathways, inducing profound oxidative and nitrosative stress, and modulating key apoptotic regulators at the transcriptional level. The synergistic interaction confirmed by both Chou–Talalay and HSA models indicates that metformin has the potential to lower the effective dose of adriamycin while maintaining or even improving therapeutic efficacy. This multimodal enhancement, together with the observed suppression of anti-apoptotic and treatment resistance associated genes, suggests that metformin may serve as a promising adjuvant agent in overcoming chemoresistance in ovarian cancer. Although further in vivo and translational studies are required, the integrated experimental and bioinformatic evidence presented here supports the potential clinical relevance of combining metformin with adriamycin to achieve more effective and potentially less toxic therapeutic strategies for ovarian cancer.

## Figures and Tables

**Table 1:** Sequence of primers used for qRT-PCR analysis (Table 1).

Gene Name	Gene Symbol	Primer Sequence (5′ → 3′)
B-cell lymphoma 2	Bcl2	F: GATGTGATGCCTCTGCGAAG R: CATGCTGATGTCTCTGGAATCT
Baculoviral IAP repeat-containing 5 (Survivin)	BIRC5	F: GGCGGGAGGATTATAATTTTCG R: CCGCCACCTCTACCAACG
BCL2-associated X protein	BAX	F: ATGTGTGTGGAGAGCGTCAA R: ACAGTTCCACAAAGGCATCC
Caspase-3	CASP3	F: GGTATTGAGACAGACAGTGG R: CATGGGATCTGTTTCTTTGC
Glyceraldehyde-3-phosphate dehydrogenase	GAPDH	F: CGGAGTCAACGGATTTGGTCGTAT R: GCCTTCTCCATGGTGGTGAAGAC
Beta-actin	ACTB	F: CCTCTGAACCCTAAGGCCAAC R: TGCCACAGGATTCCATACCC

## Data Availability

The original contributions presented in this study are included in the article. Further inquiries can be directed to the corresponding authors.
